# Effects of stimulation frequency and pulse width in transcutaneous auricular vagus nerve stimulation on finger somatosensory function

**DOI:** 10.1038/s41598-026-51309-0

**Published:** 2026-05-06

**Authors:** Kako Tanabe, Sho Kojima, Kei Saito, Hideaki Onishi

**Affiliations:** 1 Graduate School, Niigata University of Health and Welfare, Niigata, 950-3198 Japan; 2 Department of Physical Therapy, Niigata University of Health and Welfare, Niigata, 950-3198 Japan; 3 Institute for Human Movement and Medical Sciences, Niigata University of Health and Welfare, Niigata, 950-3198 Japan

**Keywords:** Transcutaneous auricular vagus nerve stimulation, Somatosensory function, Stimulation frequency, Pulse Width, Stimulation parameter, Two-point discrimination threshold, Medical research, Neurology, Neuroscience, Physiology

## Abstract

Transcutaneous auricular vagus nerve stimulation (taVNS) is a noninvasive electrical stimulation technique applied to the left cymba concha that may improve somatosensory impairment in stroke. Although previous studies have investigated various stimulation parameters, the optimal stimulation parameters for somatosensory impairment remain unknown. Therefore, the aim of this study was to determine the effective taVNS stimulation frequencies and pulse widths that enhance somatosensory function in the fingers. Experiments 1 and 2 each included 30 healthy, right-handed university students. In Experiment 1, the taVNS stimulation frequency was set to 30, 25, and 20 Hz, with a pulse width of 100 µs. In Experiment 2, the taVNS stimulation frequency was set to 25 Hz, with pulse widths of 300 µs and 500 µs. In Experiments 1 and 2, 2PD measurements were taken before taVNS, immediately after stimulation, and 20 min after stimulation ended. The results showed that Experiments 1, stimulation frequencies of 30 Hz, 25 Hz, and 20 Hz did not produce significant changes in 2PD thresholds. In contrast, Experiments 2, taVNS delivered at 25 Hz with pulse widths of 300 µs or 500 µs significantly reduced 2PD thresholds immediately after stimulation and again 20 min later.

## Introduction

Transcutaneous auricular vagus nerve stimulation (taVNS) is a noninvasive technique that delivers electrical stimulation to the cymba concha, where afferent vagus nerve fibers are distributed^1–5^. This stimulation activates the locus coeruleus (LC)—a major source of noradrenergic projections—as well as cortical and subcortical regions, including the primary sensory cortex (S1), thalamus, and insular cortex^6–8^. Consequently, taVNS can increase excitability in both the cerebral cortex and subcortical regions.

taVNS has begun to show promise as an intervention for improving somatosensory dysfunction in patients with stroke^9,10^. Although previous studies have demonstrated that stimulation parameters influence taVNS-induced effects^11^, the parameters applied in studies involving patients with stroke vary considerably^9,10^. Therefore, optimal settings—including stimulation frequency, pulse width, intensity, and duration—for improving finger somatosensory function remain unclear. Reported frequencies include 30 Hz^12^, 25 Hz ^9,13^, and 20 Hz^10,14,15^; commonly used pulse widths include 100 µs^9,13^ and 300 µs ^10,12,14,15^. Stimulation intensity is typically set at the highest level tolerable to the patient^9,10,14^ or below the pain threshold^12^, as summarized in recent reviews^16^. In studies using stroke model rats with cervical vagus nerve stimulation, the parameters frequently include a stimulation frequency of 30 Hz, a pulse width of 100 µs, and an intensity of 0.8 mA^17,18^. Thus, the stimulation parameters used for stroke patients and stroke model rats are inconsistent, and the effects of each parameter remain unclear. Therefore, we believed that in order to proceed with the application of this approach to a population of stroke patients with somatosensory dysfunction, it is first necessary to verify the effects of each stimulation parameter in healthy individuals. Therefore, based on the stimulation parameters currently used in stroke patients, we decided to conduct a parameter screening of taVNS in healthy subjects to identify stimulation parameters that effectively improve finger somatosensory function.

The aim of this study was to determine the effective taVNS stimulation frequencies and pulse widths that enhance somatosensory function in the fingers. In Experiment 1, we examined stimulation frequency because different frequencies have been shown to yield varying levels of activation in the nucleus tractus solitarius and LC^19^. Therefore, we expected that different frequencies would produce distinct effects on somatosensory function. In addition, rehabilitation has been shown to improve outcomes in stroke model rats^17,18^, so we hypothesized that stimulation at 30 Hz would produce the greatest enhancement in somatosensory function. In Experiment 2, we focused on pulse width because previous evidence indicates that LC neuronal firing rates differ depending on pulse width^20^. Given that taVNS has improved finger somatosensory function in patients with stroke^10^, we hypothesized that stimulation with a pulse width of 300 µs would yield the greatest improvement in somatosensory function.

## Materials and methods

### Study design

This study was conducted as a single-blind trial in which only the participants were blinded. Randomization was performed using the RAND function in Microsoft Excel, and no blocking was performed. Participants were not informed of the stimulation conditions to be administered. The taVNS device was fitted and stimulation was administered by the investigator. The study protocol was registered with the UMIN Clinical Trials Registry (UMIN-CTR), with the primary outcome defined as the two-point discrimination threshold and was approved on April 7, 2025 (Registration Number: UMIN000057524).

### Participants

Experiments 1 and 2 each included 30 healthy, right-handed university students (Experiment 1 [mean ± standard deviation]: 21.7 ± 1.2 years, 15 males and 15 females; Experiment 2: 22.1 ± 1.4 years, 15 males and 15 females). Handedness was assessed using the Edinburgh Handedness Test (Experiment 1: 90.6 ± 16.6; Experiment 2: 90.3 ± 15.6). Seven participants completed both experiments. In addition, the seven participants who took part in both conditions participated in the experiments after sufficient time had elapsed between them (for about 6 months). No participants reported neurological or psychiatric disorders, the use of medications affecting the central nervous system, or a history of trauma to the outer ear. The study adhered to the Declaration of Helsinki and received approval from the Ethics Review Committee of Niigata University of Health and Welfare (Approval Number: 19457–241227). All participants provided written informed consent.

### Transcutaneous auricular nerve stimulation

Experiments 1 and 2 used the tVNS^®^ R Stimulator (NEMOS, Germany). Electrode cream was applied to the two ball electrodes to improve conductivity. Stimulation was delivered to the left cymba concha, where afferent vagus nerve fibers are densely distributed^1^. Sham stimulation was applied to the left earlobe, which lacks vagus nerve innervation^15,21^. Stimulation intensity was set to 0.1 mA below each participant’s pain threshold^12^. The stimulation duration was 15 min, and the stimulation pattern was continuous^22^. The stimulation conditions in Experiment 1 included four settings: three active conditions with stimulation frequencies of 30 Hz, 25 Hz, and 20 Hz^9,10,17^, as well as one sham condition. The sham condition used a frequency of 30 Hz, which generated the highest total charge. The pulse width for all conditions was set to 100 µs^9,17^. Experiment 2 included three conditions: two active conditions with pulse widths of 300 µs and 500 µs^10,23^, as well as one sham condition. The sham condition used a pulse width of 500 µs to match the highest total charge. The stimulation frequency for all conditions in Experiment 2 was 25 Hz^9^.

### Somatosensory function assessment

In Experiments 1 and 2, somatosensory function was assessed using the two-point discrimination (2PD) test^24,25^. Measurements were obtained with a 2PD apparatus (Takei Kiki Kogyo Co., Ltd., Niigata, Japan). Participants sat in a quiet room in an upright position with a backrest, with the elbow slightly flexed. Stimuli were applied to the fingertip of the right index finger using either single or paired tactile stimuli. The stimulus pin protrusion was 1 mm, the ascent speed was 10 mm/sec, and each stimulus was presented for 1 s. Stimuli included nine two-point distances (1.0–5.0 mm in 0.5 mm increments) and a one-point stimulus (using only one pin) for a total of 10 stimulus types. In total, 160 stimuli were presented, with each stimulus type delivered 16 times in random order. Participants were instructed to respond as quickly as possible using a button in the left hand, indicating whether they perceived a single point or two points.

### Experimental protocol

**Fig. 1:**
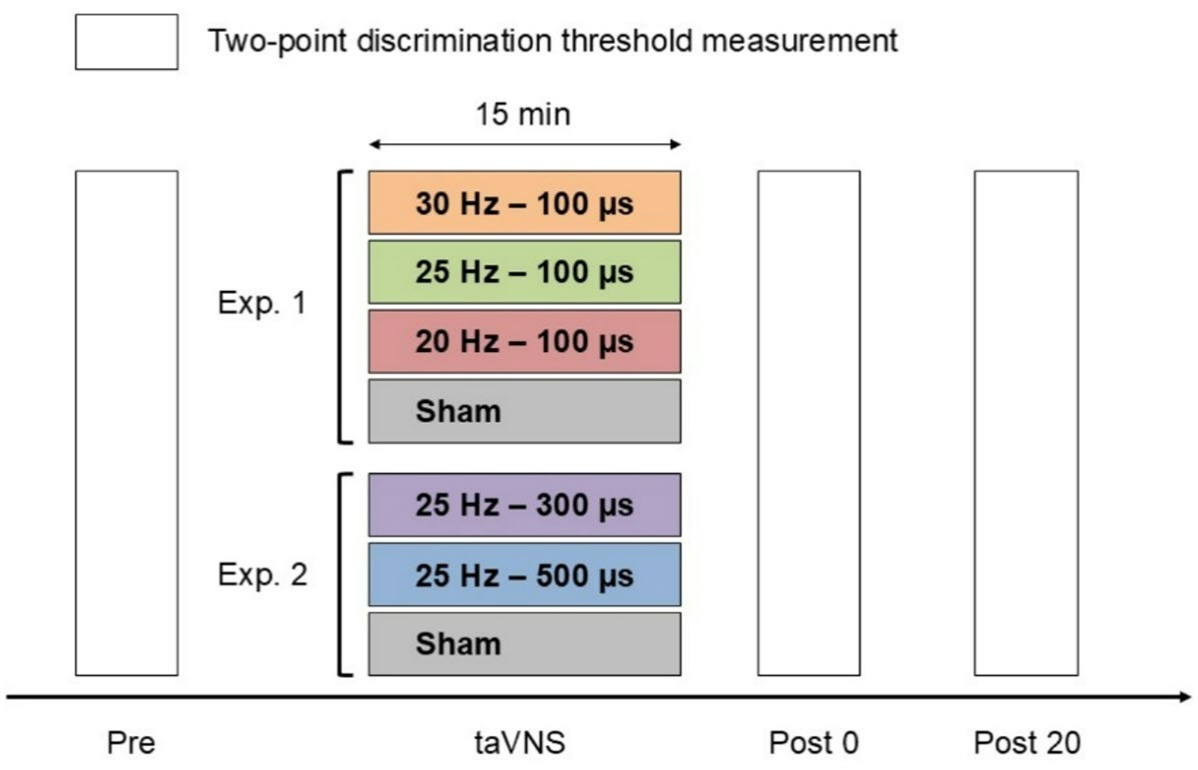
Protocol for Experiments 1 and 2. In Experiments 1 and 2, 2PD measurements were performed before taVNS (Pre), immediately after stimulation (Post 0), and 20 min after stimulation ended (Post 20). All stimulation frequency and pulse width conditions were administered with intervals of at least 5 days.

In Experiments 1 and 2, 2PD measurements were taken before taVNS (Pre), immediately after stimulation (Post 0), and 20 min after stimulation ended (Post 20). In Experiment 1, taVNS was applied for 15 min under one of three frequency conditions—30 Hz, 25 Hz, or 20 Hz—or under a sham condition. In Experiment 2, taVNS was applied for 15 min under one of two pulse width conditions—300 µs or 500 µs—or under a sham condition (Fig. 1). In both experiments, all frequency and pulse width conditions were administered to each participant in randomized order with intervals of at least 5 days between sessions.

### Data analysis

The 2PD data were analyzed in MATLAB (The MathWorks, Inc.). Correct response rates were plotted on the vertical axis against interpoint distances on the horizontal axis. Logistic regression using a generalized linear model was performed, and the 2PD threshold was defined as the interpoint distance corresponding to a 50% correct response rate^25^.

### Statistical analysis

Statistical analyses were conducted using SPSS Statistics 27 (IBM, Armonk, NY, USA). Based on previous study^20^, the total charge (nC) for taVNS in Experiments 1 and 2 was calculated as the product of stimulation intensity (mA), pulse width (ms), stimulation frequency (Hz), and stimulation duration (s). Total charge was compared across taVNS conditions using the Kruskal–Wallis test, with Bonferroni-adjusted Post hoc comparisons. The 2PD thresholds were analyzed using repeated-measures two-way ANOVA.

Experiment 1: Condition (30 Hz, 25 Hz, 20 Hz, sham) × Time (Pre, Post 0, Post 20).

Experiment 2: Condition (300 µs, 500 µs, sham) × Time (Pre, Post 0, Post 20).

Bonferroni corrections were applied to all Post hoc tests. The significance level was set at 5%.

## Results

The stimulation intensities and total charges delivered in Experiments 1 and 2 are shown in Table 1. The Kruskal–Wallis test indicated a significant difference in total charge across conditions (*p* < 0.001).

**Table 1:** Comparison of stimulus intensity and total charge in experiments 1 and 2.

	Condition	Stimulus intensity (mA)	Frequency (Hz)	Pulse width (µs)	Stimulation time (sec)	Total charge amount (nC)
Exp. 1	30 Hz–100 µs	1.61 ± 1.14	30	100	900	4,338,000 ± 3065765.16
	25 Hz–100 µs	1.53 ± 0.95	25	100	900	3,450,000 ± 2140327.08
	20 Hz–100 µs	1.42 ± 0.78	20	100	900	2,550,000 ± 1408211.63
	Sham	1.98 ± 0.98	30	100	900	5,346,000 ± 2643978.82
Exp. 2	25 Hz–300 µs	1.28 ± 0.42	25	300	900	8,617,500 ± 2830085.03
	25 Hz–500 µs	0.89 ± 0.46	25	500	900	9,975,000 ± 5202102.94
	Sham	1.18 ± 0.46	25	500	900	13,237,500 ± 5201697.44
					*mean ± standard division	

	Comparison of total charge amount				*p*-value	
Exp. 1	30 Hz–100 µs vs. 25 Hz–100 µs				*p* = 1.000	
	30 Hz–100 µs vs. 20 Hz–100 µs				*p* = 0.935	
	25 Hz–100 µs vs. 20 Hz–100 µs				*p* = 1.000	
	30 Hz–100 µs vs. Sham				*p* = 1.000	
	25 Hz–100 µs vs. Sham				*p* = 0.409	
	20 Hz–100 µs vs. Sham				***p*** **= 0.012**	
Exp. 2	25 Hz–300 µs vs. 25 Hz–500 µs				*p* = 1.000	
	25 Hz–300 µs vs. Sham				*p* = 0.959	
	25 Hz–500 µs vs. Sham				*p* = 1.000	
Exp. 1 vs. Exp. 2	30 Hz–100 µs vs. 25 Hz–300 µs				***p*** **< 0.001**	
	30 Hz–100 µs vs. 25 Hz–500 µs				***p*** **< 0.001**	
	25 Hz–100 µs vs. 25 Hz–300 µs				***p*** **< 0.001**	
	25 Hz–100 µs vs. 25 Hz–500 µs				***p*** **< 0.001**	
	20 Hz–100 µs vs. 25 Hz–300 µs				***p*** **< 0.001**	
	20 Hz–100 µs vs. 25 Hz–500 µs				***p*** **< 0.001**	

**Fig. 2:**
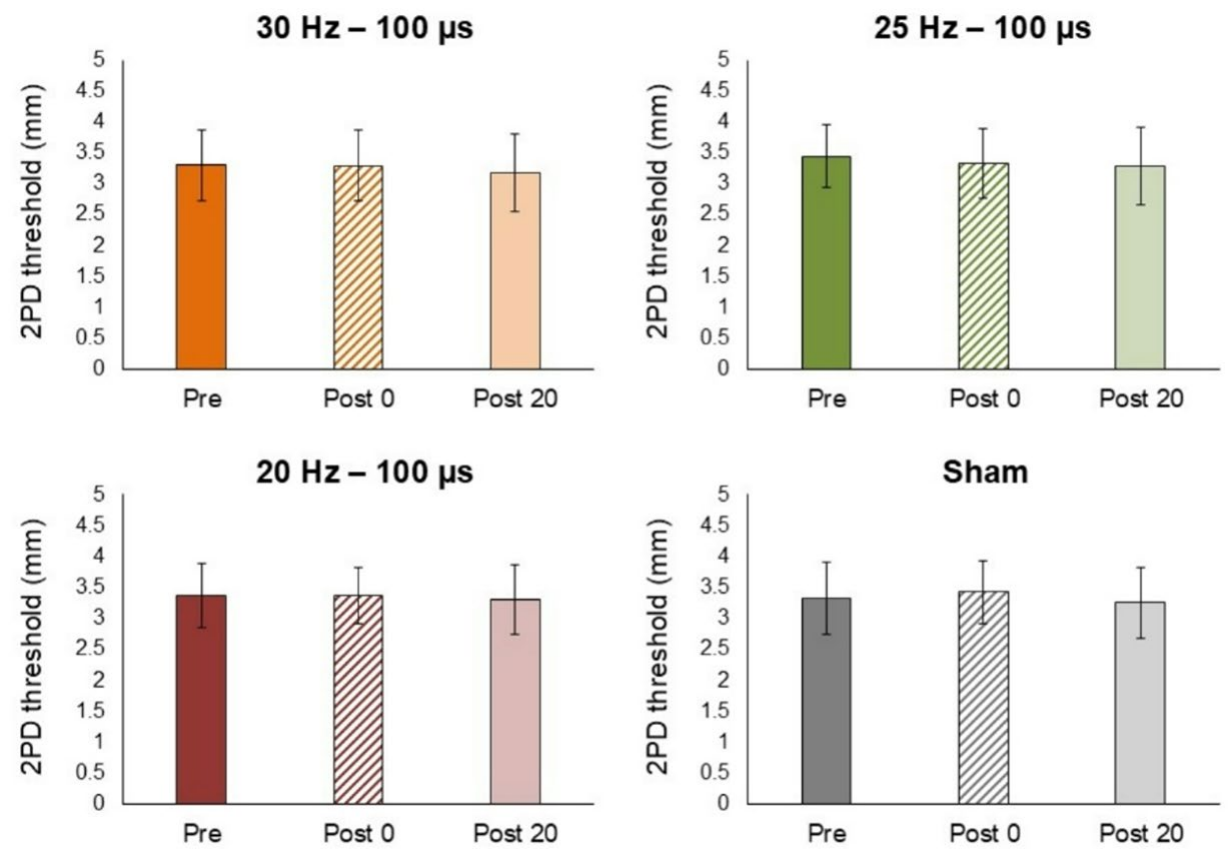
Effects of taVNS frequency conditions on the 2PD threshold. Changes in 2PD thresholds were compared across frequency conditions at Pre, Post 0, and Post 20. No significant condition × time interaction was observed for any frequency condition.

Figure 2 presents the 2PD thresholds obtained in Experiment 1. Under the 30 Hz condition, thresholds were 3.30 ± 0.57 mm (Pre), 3.29 ± 0.57 mm (Post 0), and 3.18 ± 0.62 mm (Post 20). Under the 25 Hz condition, thresholds were 3.44 ± 0.51 mm (Pre), 3.32 ± 0.56 mm (Post 0), and 3.28 ± 0.62 mm (Post 20). For the 20 Hz condition, thresholds were 3.37 ± 0.53 mm (Pre), 3.37 ± 0.45 mm (Post 0), and 3.31 ± 0.57 mm (Post 20). In the sham condition, thresholds were 3.32 ± 0.58 mm (Pre), 3.43 ± 0.50 mm (Post 0), and 3.25 ± 0.56 mm (Post 20). Repeated-measures two-way ANOVA showed no main effect of condition (F(3, 87) = 0.575, *p* = 0.633, partial η² = 0.019) and no condition × time interaction (F (6, 174) = 1.245, *p* = 0.292, partial η² = 0.041). On the other hand, a main effect of the time factor (F(2,58) = 3.596, *p* = 0.042, partial η² = 0.110) was observed; however, post hoc tests revealed no significant differences within the time factor (Pre-Post 0, *p* = 1.000, 95% CI [-0.082, 0.093]; Pre-Post 20, *p* = 0.160, 95% CI [-0.027, 0.233]; Post 0–Post 20, *p* = 0.086, 95% CI [-0.010, 0.206]).

**Fig. 3:**
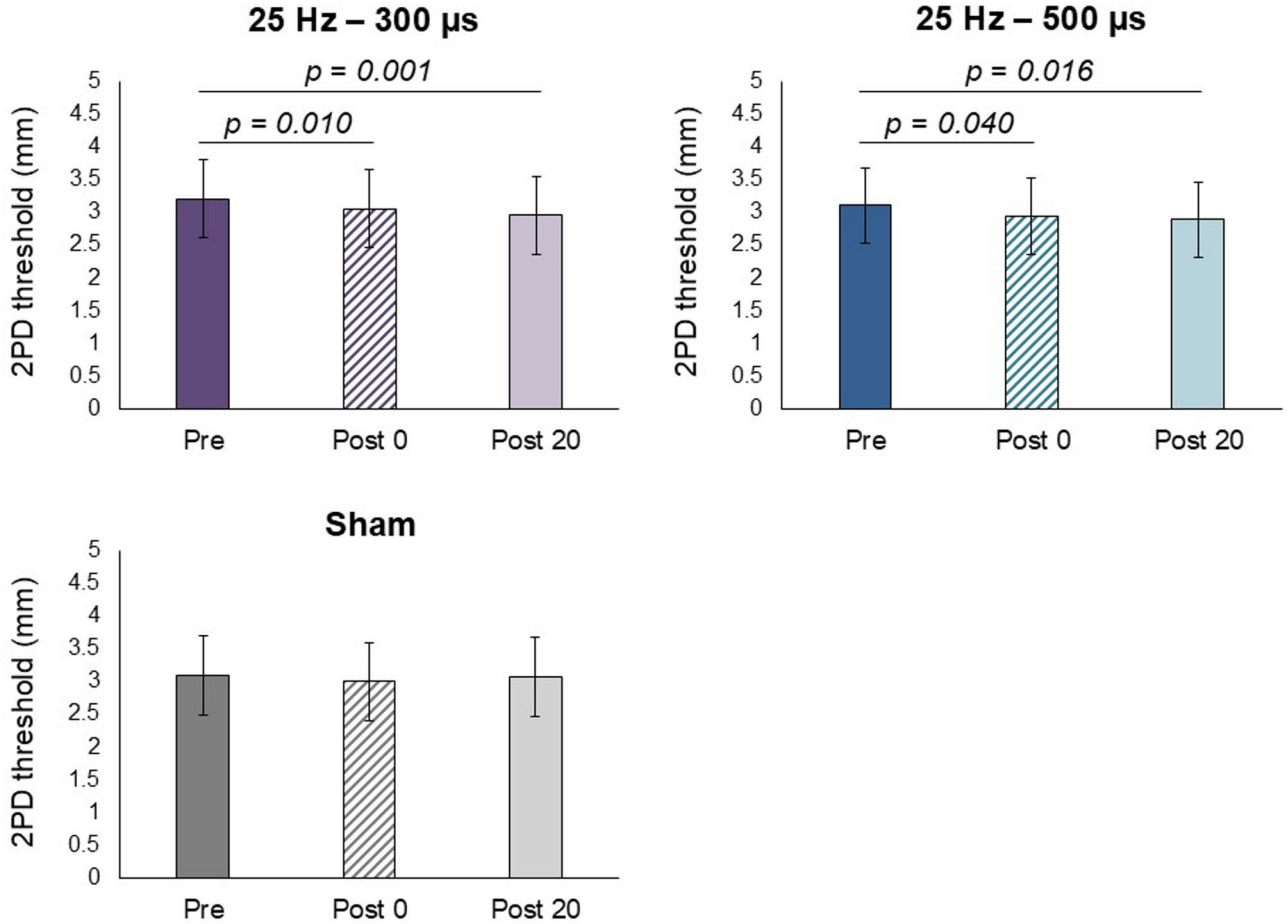
Effects of pulse width conditions on the 2PD threshold. Changes in 2PD thresholds were compared across pulse width conditions at Pre, Post 0, and Post 20. Under the 300 µs condition, 2PD thresholds were significantly reduced at Post 0 and Post 20 compared with Pre (Pre vs. Post 0, *p* = 0.010; Pre vs. Post 20, *p* = 0.001; Post 0 vs. Post 20, *p* = 0.373). Similarly, under the 500 µs condition, thresholds were significantly reduced at Post 0 and Post 20 (Pre vs. Post 0, *p* = 0.040; Pre vs. Post 20, *p* = 0.016; Post 0 vs. Post 20, *p* = 1.000). In contrast, no significant changes were observed in the sham condition (Pre vs. Post 0, *p* = 0.465; Pre vs. Post 20, *p* = 1.000; Post 0 vs. Post 20, *p* = 1.000).

Figure 3 shows the 2PD thresholds obtained in Experiment 2. Under the 300 µs condition, thresholds were 3.21 ± 0.59 mm (Pre), 3.06 ± 0.56 mm (Post 0), and 2.95 ± 0.61 mm (Post 20). Under the 500 µs condition, thresholds were 3.10 ± 0.57 mm (Pre), 2.94 ± 0.54 mm (Post 0), and 2.89 ± 0.49 mm (Post 20). In the sham condition, thresholds were 3.09 ± 0.60 mm (Pre), 3.00 ± 0.68 mm (Post 0), and 3.07 ± 0.61 mm (Post 20). Repeated-measures two-way ANOVA revealed no main effect of condition (F(2, 58) = 0.970, *p* = 0.385, partial η² = 0.032). In contrast, there was a significant main effect of time (F(2, 58) = 8.543, *p* = 0.001, partial η² = 0.228) and a significant condition × time interaction (F(4, 116) = 2.578, *p* = 0.041, partial η² = 0.082). Post hoc comparisons revealed that under the 300 µs condition, 2PD thresholds were significantly lower at Post 0 and Post 20 compared to Pre (Pre vs. Post 0, *p* = 0.010, 95% CI [0.031, 0.271]; Pre vs. Post 20, *p* = 0.001, 95% CI [0.091, 0.417]; Post 0 vs. Post 20, *p* = 0.373, 95% CI [-0.062, 0.269]). Similarly, under the 500 µs condition, thresholds were also significantly reduced at Post 0 and Post 20 (Pre vs. Post 0, *p* = 0.040, 95% CI [0.006, 0.324]; Pre vs. Post 20, *p* = 0.016, 95% CI [0.034, 0.397]; Post 0 vs. Post 20, *p* = 1.000, 95% CI [-0.091, 0.192]). In contrast, no significant changes were observed in the sham condition (Pre vs. Post 0, *p* = 0.465, 95% CI [-0.068, 0.251]; Pre vs. Post 20, *p* = 1.000, 95% CI [-0.133, 0.181]; Post 0 vs. Post 20, *p* = 1.000, 95% CI [-0.242, 0.107]). Furthermore, comparisons of Pre stimulation thresholds across conditions showed no significant differences (300 µs vs. 500 µs, *p* = 0.804; 300 µs vs. sham, *p* = 0.477; 500 µs vs. sham, *p* = 1.000).

Regarding side effects of taVNS, in neither Experiment 1 nor Experiment 2 did any participants report physical adverse events during or after taVNS stimulation.

## Discussion

This study examined the effects of stimulation frequency and pulse width in taVNS on somatosensory function in the fingers. The results showed that stimulation frequencies of 30 Hz, 25 Hz, and 20 Hz did not produce significant changes in 2PD thresholds. In contrast, taVNS delivered at 25 Hz with pulse widths of 300 µs or 500 µs significantly reduced 2PD thresholds immediately after stimulation and again 20 min later.

The stimulation frequencies selected in Experiment 1 were based on previous studies involving patients with stroke and stroke model rats^9,10,12–14,17,18^. One possible explanation is the influence of additional stimulation parameters, particularly pulse width. In healthy adults, taVNS at 25 Hz and 250 µs has been shown to increase activation in S1 compared with sham stimulation^8^. Furthermore, a comparison between a group of stroke patients who received 25 Hz, 500 µs taVNS and a sham stimulation group showed that cerebral blood flow in the sensorimotor cortex increased in the taVNS group^23^. The pulse widths used in previous studies that demonstrated S1 activation and increased cerebral blood flow in the sensorimotor cortex were longer than the 100 µs pulse width applied in this study. In addition to pulse width, stimulation duration may also influence taVNS effectiveness. For example, applying taVNS for 20 min has been shown to improve somatosensory function in patients with stroke^9^, and administering taVNS for 1 h in combination with rehabilitation has improved motor function impairments^12,15^. Taken together, these findings suggest that the total amount of stimulation delivered in Experiment 1—due to the short pulse width and relatively brief stimulation duration—may have been insufficient to alter the 2PD threshold, regardless of stimulation frequency.

In Experiment 2, the pulse widths were increased to 300 µs and 500 µs. The total stimulation delivered under the 300 µs and 500 µs conditions was also significantly greater than that delivered under the 30 Hz, 25 Hz, and 20 Hz conditions in Experiment 1, where no changes in 2PD thresholds were observed. Previous studies in rats support these findings: larger pulse widths during LC stimulation produce higher LC neuronal firing rates^20^, and increasing pulse width during vagus nerve stimulation leads to greater pupillary dilation^5,15^, an indirect indicator of noradrenaline release from the LC. In addition, combining taVNS with pulse widths of 300 µs or 500 µs and rehabilitation therapy improves upper limb motor and sensory function in patients with stroke^10,12,14,15^. Beyond somatosensory outcomes, prior studies have shown that taVNS at 25 Hz and 500 µs enhances spatial working memory in healthy individuals^26^ and that taVNS at 25 Hz and 250 µs reduces reaction times to target stimuli^27^. These findings indicate that studies reporting improvements in bodily functions frequently used pulse widths longer than 100 µs. Moreover, taVNS has been shown to increase heart rate variability in a charge-dependent manner^28^, suggests that the pulse width used in Experiment 2 likely provided sufficient stimulation to lower the 2PD threshold. Overall, the present findings indicate that effective enhancement of somatosensory function with taVNS occurs when using 25 Hz in combination with pulse widths of 300 µs and 500 µs, conditions that likely provide adequate stimulation intensity. These results suggest that applying a specific level of electrical stimulation via taVNS may improve somatosensory function in the fingers. Furthermore, the magnitude of change in the 2PD threshold observed in this study was consistent with that reported in previous studies using peripheral tactile stimulation interventions^24^. However, further investigation is required to determine whether the changes in the 2PD threshold associated with the stimulation parameters that improved somatosensory function in this study are also observed in stroke patients, and whether these changes are functionally meaningful.

This study has several limitations. First, since no prior power analysis was conducted based on the sample size, the statistical results must be interpreted with caution. Second, this study did not examine the combined effects of both stimulation frequency and pulse width. Consequently, it remains unclear in Experiment 2 which factor—stimulation frequency or pulse width—contributed to the improvement in somatosensory function. Third, since the taVNS stimulation intensity in this study was tailored to each participant, the changes in somatosensory function observed may have been influenced by the stimulation intensity; therefore, future studies should validate the parameters while accounting for the influence of stimulation intensity. Fourth, since participants in this study were not asked whether the administered stimulation was real or sham, it is unclear whether blinding was achieved. Fifth, since physiological markers were not measured in this study, it is unclear what neurological mechanisms underlie the changes in somatosensory function. Therefore, in future studies, it will be necessary to verify the mechanisms underlying the stimulation effects by measuring pupil diameter—which indirectly indicates norepinephrine release induced by taVNS^5^—and by evaluating the excitability of S1 using an electroencephalograph.

## Conclusion

In conclusion, taVNS delivered at 30 Hz, 25 Hz, or 20 Hz with a pulse width of 100 µs did not alter somatosensory function in the fingers. In contrast, taVNS at 25 Hz combined with longer pulse widths of 300 µs and 500 µs significantly improved somatosensory function following stimulation.

## Data Availability

The raw data supporting the conclusions of this study are available from the corresponding author upon reasonable request.
